# Spatiotemporal characteristics of pandemic influenza

**DOI:** 10.1186/1471-2334-14-378

**Published:** 2014-07-09

**Authors:** Lars Skog, Annika Linde, Helena Palmgren, Hans Hauska, Fredrik Elgh

**Affiliations:** 1 Division of Geodesy and Geoinformatics, Department of Urban Planning and Environment, Royal Institute of Technology (KTH), SE-100 44, Stockholm, Sweden; 2 Public Health Agency of Sweden, SE-100 44, Solna, Sweden; 3 Department of Clinical Microbiology, Umeå University, SE-100 44, Umeå, Sweden

**Keywords:** Russian influenza, Asian influenza, A (H1N1) pdm09, Spatiotemporal spread, Temperature dependence, Spatial modelling, GIS

## Abstract

**Background:**

Prediction of timing for the onset and peak of an influenza pandemic is of vital importance for preventive measures. In order to identify common spatiotemporal patterns and climate influences for pandemics in Sweden we have studied the propagation in space and time of A(H1N1)pdm09 (10,000 laboratory verified cases), the Asian Influenza 1957–1958 (275,000 cases of influenza-like illness (ILI), reported by local physicians) and the Russian Influenza 1889–1890 (32,600 ILI cases reported by physicians shortly after the end of the outbreak).

**Methods:**

All cases were geocoded and analysed in space and time. Animated video sequences, showing weekly incidence per municipality and its geographically weighted mean (GWM), were created to depict and compare the spread of the pandemics. Daily data from 1957–1958 on temperature and precipitation from 39 weather stations were collected and analysed with the case data to examine possible climatological effects on the influenza dissemination.

**Results:**

The epidemic period lasted 11 weeks for the Russian Influenza, 10 weeks for the Asian Influenza and 9 weeks for the A(H1N1)pdm09. The Russian Influenza arrived in Sweden during the winter and was immediately disseminated, while both the Asian Influenza and the A(H1N1)pdm09 arrived during the spring. They were seeded over the country during the summer, but did not peak until October-November. The weekly GWM of the incidence moved along a line from southwest to northeast for the Russian and Asian Influenza but northeast to southwest for the A(H1N1)pdm09. The local epidemic periods of the Asian Influenza were preceded by falling temperature in all but one of the locations analysed.

**Conclusions:**

The power of spatiotemporal analysis and modeling for pandemic spread was clearly demonstrated. The epidemic period lasted approximately 10 weeks for all pandemics. None of the pandemics had its epidemic period before late autumn. The epidemic period of the Asian Influenza was preceded by falling temperatures. Climate influences on pandemic spread seem important and should be further investigated.

## Background

An early understanding of factors allowing for efficient spread of a new influenza virus is vital for the handling of a pandemic. Correct predictions of the onsets of epidemic periods in different areas may allow for optimal distribution of vaccines, antivirals and of social distancing, whereby the consequences of the outbreak may be mitigated. Pre-existing immunity, social behaviour and, most likely, climate factors [1,2] are major determinants for the spread of pandemic influenza viruses. The propagation of an influenza is also dependent on certain traits of the different viruses and possibly also by interference from other viruses [3,4], which may be more efficiently spread than the influenza virus under certain climate conditions [5]. As a result the R_0_-value [6] may vary for the same influenza virus.

Studies on the spread of viruses among humans and results obtained from guinea pig experiments under different climate conditions [7,8] may facilitate the understanding of viral traits affecting the dissemination. However, we can also get new knowledge from old pandemics. To better understand factors governing the dissemination of pandemic viruses we have investigated the spread patterns for three past influenza pandemics in Sweden, using spatiotemporal analysis, spatial modeling and visualization. The study is based on reported case data from the Russian Influenza in 1889–90 [9-12], the Asian Influenza in 1957–58 [13] (An animated map of the global spread can be found in Additional file 1) and the A(H1N1) pdm2009-2010 [14-17]. The paper also discusses possible co-variation of influenza dissemination with temperature and precipitation for the years 1957–1958.

## Methods

### Data acquisition and preparation

In the beginning of 1890, shortly after the outbreak of the *Russian Influenza* in Sweden, all Swedish physicians were asked by the Swedish Society of Medicine to provide information about the start and the peak of the pandemic and the total number of cases in their region. They were also asked to fill in a questionnaire on the number, sex and age of infected persons in the households they visited. General answers on the epidemic were received from 398 physicians and data on individual patients were available for more than 32,600 persons [10] starting from the first week of December 1899. The information was compiled into a table, providing detailed information on the development of the influenza at 69 locations.

In 1957, when the *Asian Influenza* arrived, the Royal Medical Board [18] asked the district physicians throughout the country to report on clinically diagnosed influenza cases on a weekly basis. The reports on diagnosed cases, provided by 713 out of 720 district physicians during the period of June 1957 to February 1958 [19], have been stored in the Swedish National Archives [20]. For this study the weekly reports (1200 pages) were scanned into digital documents. From the scanned pages we entered location of the physicians, date and the weekly number of infected cases into an Excel spread sheet. In total 275,000 cases were recorded.

For the period of the Asian Influenza in Sweden we also acquired daily observations of temperature and precipitation from 39 weather stations. The data was made available by the Swedish Meteorological and Hydrological Institute [21]. For the years 1957–58 there are unfortunately no data on humidity available for Sweden.

Approximately 60 infectious diseases are continuously surveyed by the Swedish Institute for Communicable Disease Control [22] (now Public Health Agency of Sweden [23]) through statutory notifications according to the Communicable Disease Act. From 15 May, 2009 and onwards all laboratory verified *Influenza A(H1N1)pdm2009-2010* (hereafter called A(H1N1)pdm09) cases have been reported in the web-based reporting system SmiNet [24]. Up-to-date census data per municipality are published each year by Statistics Sweden [25] and historic records on population (for the time of the Russian and the Asian Influenza) have been collected and made available to researchers at the Demographic Data Base [26] at Umeå University. The present municipality division, established in 2003, was used to enable comparisons over time of the influenza data from the three pandemics.

No ethical approval was required to access the data for the study.

### Geocoding

The case tables from the *Russian Influenza* were checked for inconsistencies and some of the place names were changed to the spelling of today. The data was converted into Excel format, to enable geocoding. In ArcGIS [27] the tables were spatially joined to the municipalities with reported cases. The weekly number of cases was assigned to each municipality according to the present municipality division.

**Figure 1:**
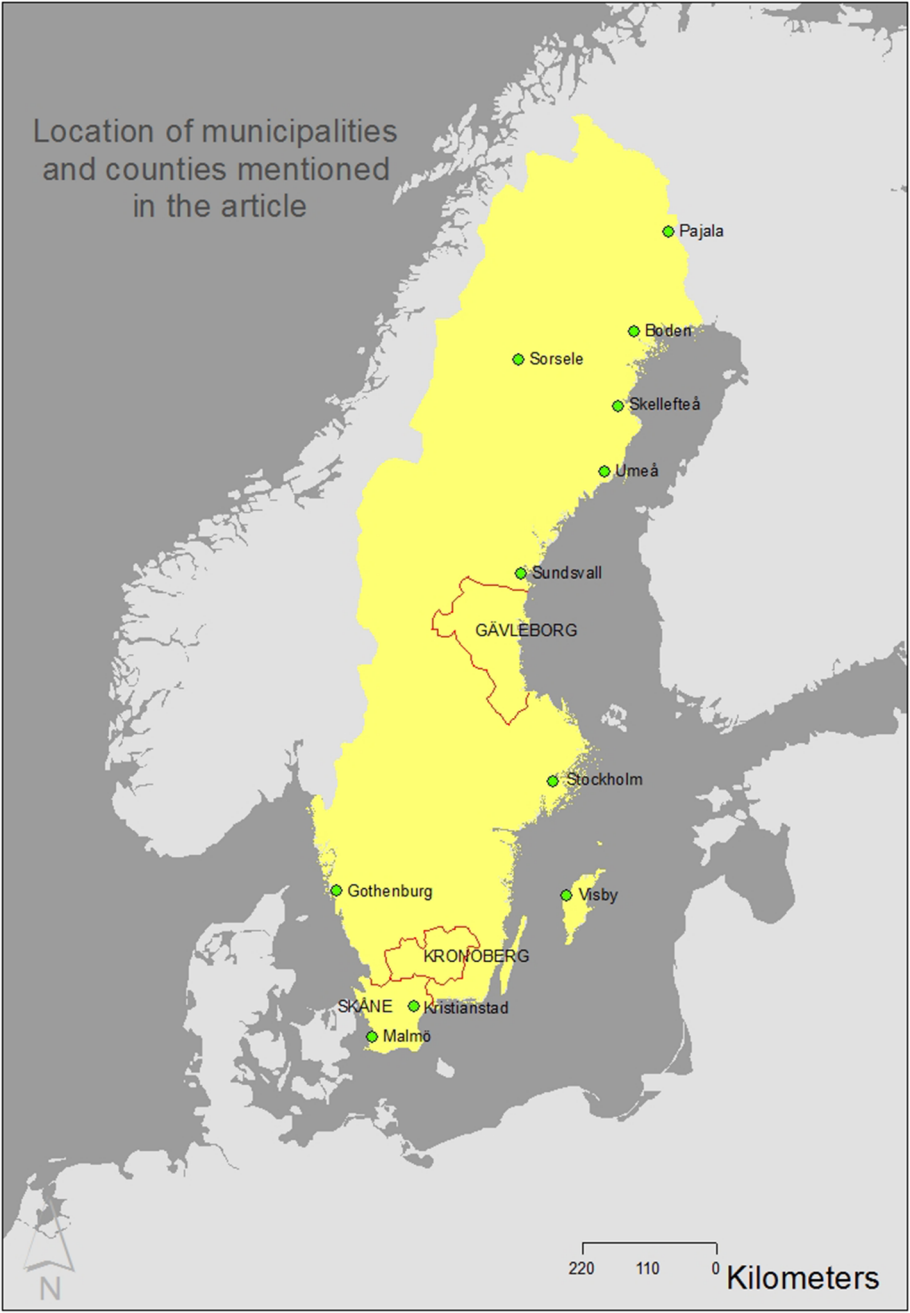
**Locations mentioned in the article.** The map indicates the centroids for municipalities mentioned in the article as well as the county limits for the counties Gävleborg, Kronoberg and Skåne.

For the *Asian Influenza* a table was created where each weekly report was assigned coordinates [28] for the locations of the cases. Geocoding was performed using the “Find Coordinates” function in the Swedish place finder service hitta.se [29]. ArcGIS was used to create geographic layers from the tabular data with one row for each report (number of diagnosed cases per week, reported per district physician). Reports are available from 516 districts. All in all there are 5538 such reports on the number of diagnosed cases per location. The reports cover 29 weeks, starting 9 July, 1957 and ending 31 January, 1958. The total number of diagnosed cases is 276,537. Detailed data are lacking for the counties of Gävleborg, Kronoberg and Skåne (As a service to non-Swedish readers of this article we have created a simple map (Figure 1) indicating locations mentioned in this work). For these counties, representing 3.9, 1.2 and 11.4% of the Swedish population in 1957 respectively, all available data has been assigned to their respective administrative centers. For unknown reasons the case data reporting for the Stockholm area is incomplete.

The geocoding for the *A(H1N1)pdm09* is based on the 10077 laboratory verified cases with identifiable postal codes in SmiNet reported between 1 June, 2009 and 8 March, 2010. In SmiNet it is mandatory to report national registration numbers. As the address information in some cases was incomplete, PAR AB [30], a company specialized in supplying address information for commercial purposes, was engaged to match the incomplete cases with a database called SPAR [31]. The database contains address and other information for all individuals in Sweden with a national registration number. For this study, it was decided to geocode at postal code level only, in order not to violate Swedish laws on personal integrity. Geocoding was performed in ArcGIS, connecting the case data with the centroids of the postal code areas and with their respective municipality.

### Climate factors (Asian influenza)

From the daily observations of temperature and precipitation we calculated the mean temperature and the accumulated precipitation for each week of the duration of the Asian Influenza pandemic in Sweden (i.e. the period for which we have data on infected persons). The meteorological data was combined with the case data in two ways. First we combined the weekly weather observations with the case data from the eight major cities (from Malmö in the South to Luleå in the North representing 24.4% of the population in 1957) where weather stations were located (the rest of the 39 weather stations were located in places with very few inhabitants). Least squares linear regression was used to calculate trend lines for temperature for the weeks prior to the onset of epidemic periods. Weekly z-score values ((observation-mean value)/standard deviation) for temperature, precipitation and number of cases were used to create diagrams to study the impact of temperature and precipitation on the local number of influenza cases. Furthermore, we used the spatial statistics functions in ArcGIS to interpolate (inverse distance) weekly weather data for all locations with diagnosed cases of influenza in order to determine whether there was any correlation between the number of cases and temperature and/or precipitation.

### Duration of epidemic periods and spatial distribution of peaking week

We developed a new method to estimate and compare the duration of the epidemic periods for the three pandemics. First, z-score values for the weekly number of cases for each pandemic were calculated, disregarding whether the number of cases were based on clinical observations or if they were laboratory verified. We then defined the start of the epidemic period as the first week when the slope of the graph for the z-scores exceeded 20, provided that it did so for three consecutive weeks. Using this technique we identified the onset of the pandemic period for all (21) county capitals. The end of the epidemic period was defined as the week when the slope, after a decline in the number of cases to approximately the same level as before the start of the peak, was exceeding −10 for three consecutive weeks. We also created diagrams of the z-score values for the weekly number of cases.

The peaking week for each municipality was identified and added as an attribute to the municipality feature class. Using this data we had new means to statistically study the spatiotemporal characteristics of the pandemics. We created maps for the three pandemics and Moran’s I was used to describe the pattern of peaking weeks. Spatial correlation between latitude and peaking week was also investigated.

### Spatiotemporal visualization

Maps were created in ArcGIS displaying the geographical spread of the three pandemics, week by week in total numbers as well as incidence per municipality (cumulative number of cases per week divided by number of inhabitants). Coordinates for the geographically weighted mean (GWM) for weekly incidence per municipality were also calculated, using formula (1).

(1) $$\bar{X_{w}}=\frac{\sum_{i=1}^{n}w_{i}x_{i}}{\sum_{i=1}^{n}w_{i}}\;,\;\bar{Y_{w}}=\frac{\sum_{i=1}^{n}w_{i}y_{i}}{\sum_{i=1}^{n}w_{i}},\;$$

where **w**_**i**_ is the incidence in municipality i.

The weekly maps were converted into video animations. From the weekly GWM locations for the epidemic period geographic vectors were created and analyzed to establish a statistical mean direction for each of the three moving patterns of the GWM.

## Results

**Figure 2:**
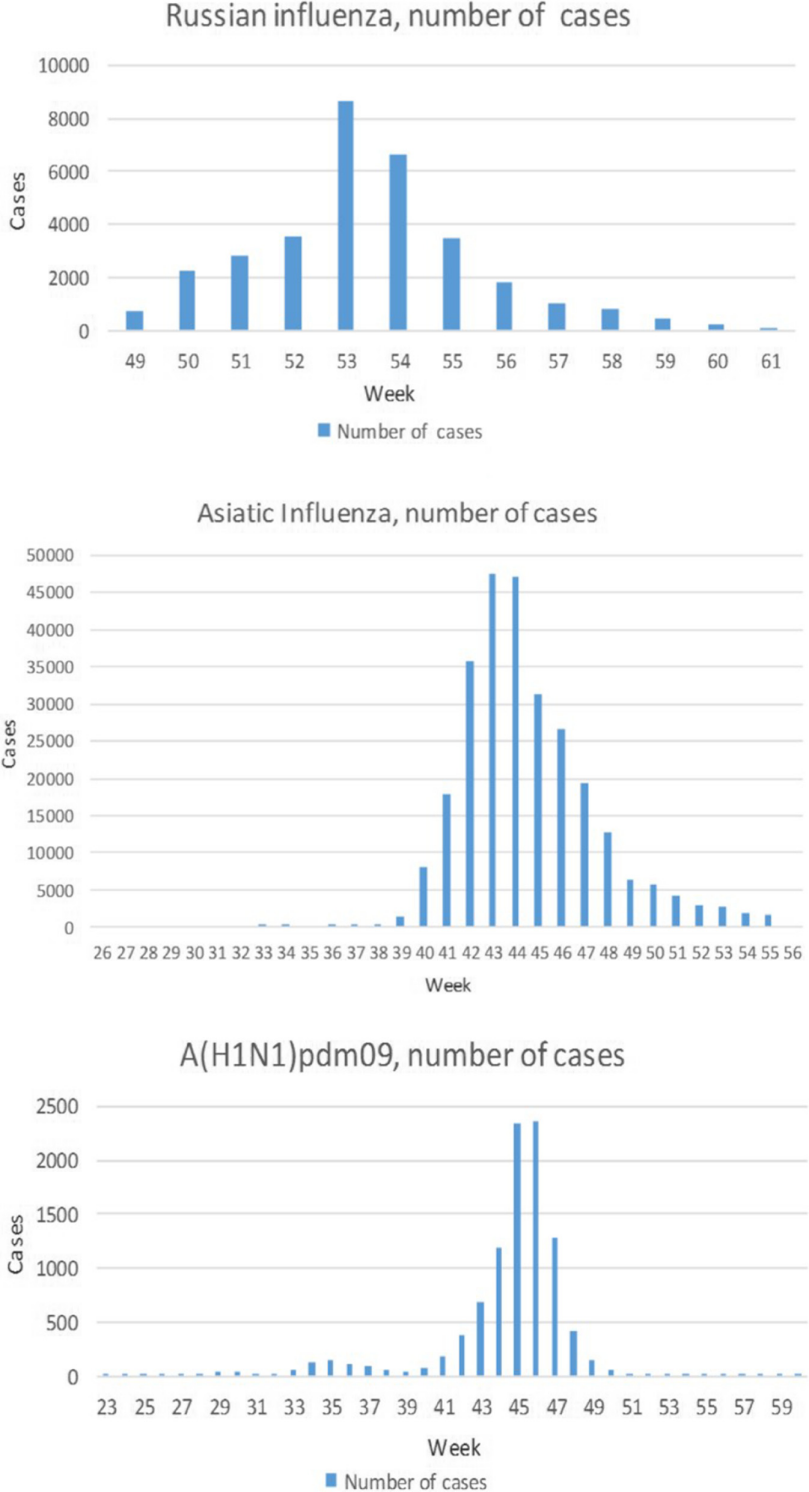
**Distribution in time for the three pandemics.** These diagrams show the distribution in time of absolute numbers of ILI cases for the Russian and the Asian Influenza and laboratory verified cases for the A(H1N1)pdm09).

**Figure 3:**
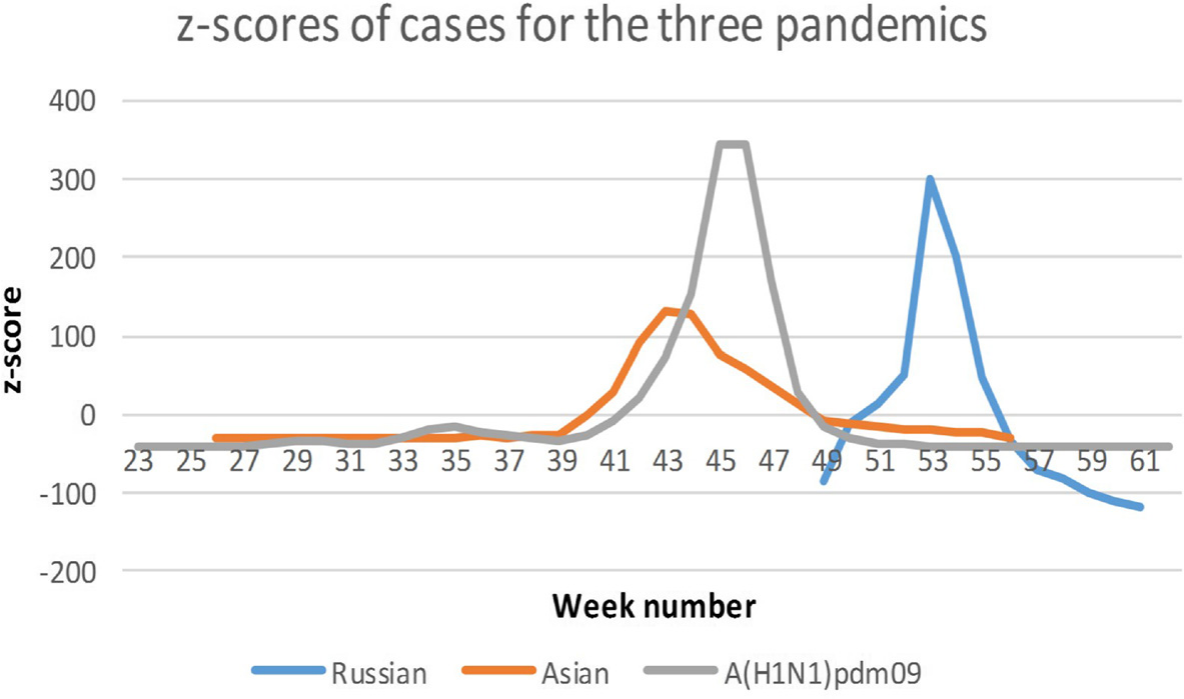
**z-scores for the values presented in Figure**2**(distribution in time of absolute numbers of ILI cases for the Russian and the Asian influenza and laboratory verified cases for the A(H1N1)pdm09).**

**Figure 4:**
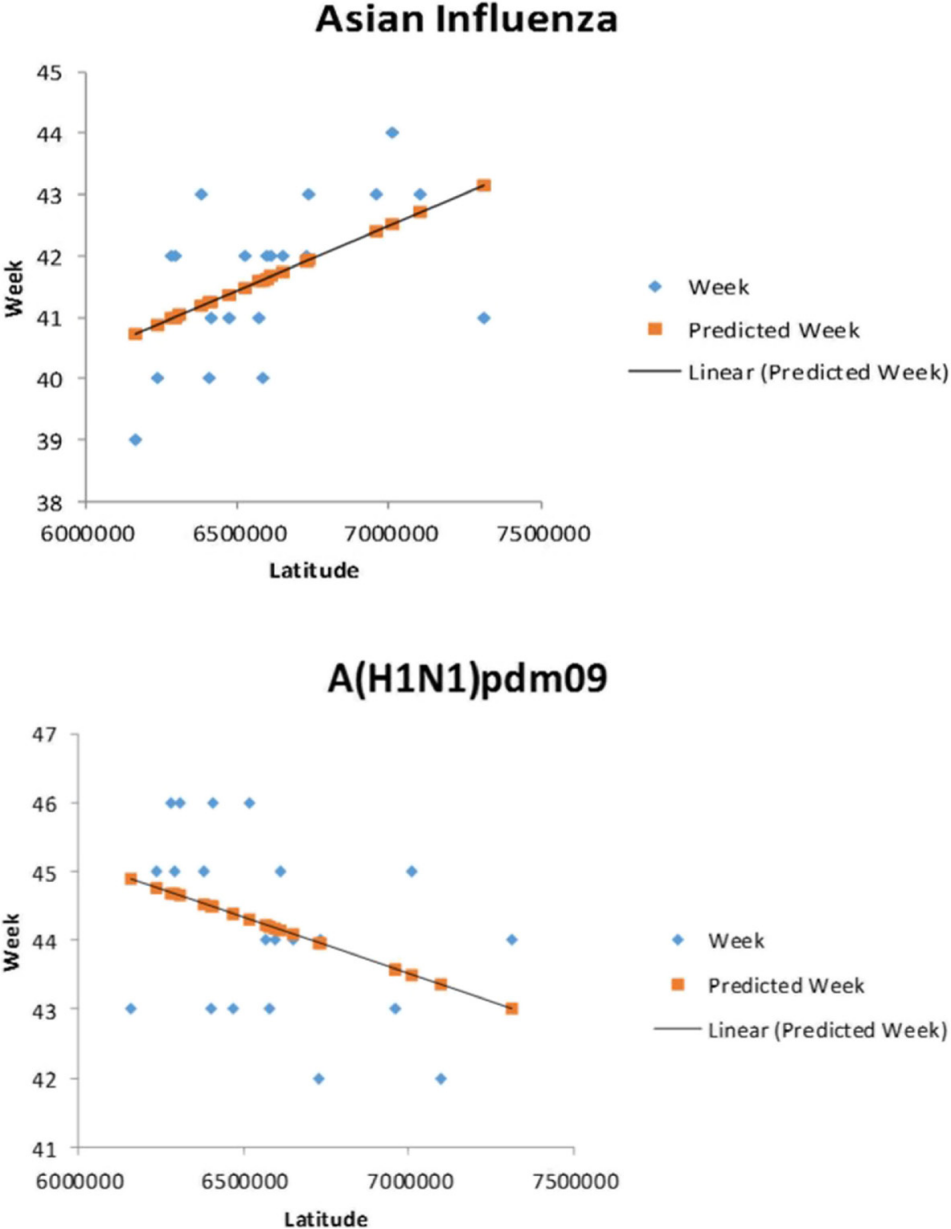
**Time for onset of peaking period vs latitude.** The two graphs depict the association between time for onset of peaking period vs latitude for the 21 county capitals of Sweden for the Asian Influenza and the A(H1N1)pdm09. Standard error and p-value for the predicted trend line are 1.09 and 0.017 respectively for the Asian and 1.22 and 0.087 for the A(H1N1)pdm09.

The distribution in time of the pandemics is shown in Figures 2 and 3. The animated maps in Additional files 2, 3 and 4 depict spatiotemporal incidence and GWM for the Russian Influenza, the Asian Influenza and the A(H1N1)pdm09 respectively. The weekly GWM of the incidence moved along a line from southwest to northeast for the Russian and the Asian influenza, but northeast to southwest for the A(H1N1)pdm09. The correlation analysis for the time of the onset of the peaking period vs latitude for the county capitals for the Asian Influenza and the A(H1N1)pdm09 (Figure 4) vaguely supports the results of the GWM analysis. Using the definitions for start and end of epidemic periods, it was found that the Russian Influenza, the Asian Influenza and the A(H1N1)pdm09 lasted 11, 10 and 9 weeks respectively (as illustrated in Figure 3) and the proportion of cases during these weeks were 99.2, 90.6 and 89.5 respectively ( Table 1).

**Table 1:** Number of cases for the three pandemics

**Pandemic**	**Total number of cases**	**Cases within 10 weeks epidemic period**				**Total number of weeks**
		**Number**	**%**	**Mean**	**STDV**	
Russian Influenza	32,642	32,366	99.2	2511	21	13
Asian Influenza	276,537	250,518	90.6	8921	297	30
A(H1N1)pdm09	10,077	9,203	89.5	252	6	40

The table shows the total number of influenza cases reported for the three pandemics, as well as the numbers and percentages for the cases within the 10 weeks epidemic period. The A(H1N1)pdm09 cases were all laboratory verified.

Skog et al.

### Russian influenza

The Russian influenza outbreak started in the Stockholm region in November 1889. Data are available from the first week of December 1889 (week 8949) when the incidence was already high in the Stockholm region and isolated cases were found in many places along the railroad network from Malmö in the south to Sundsvall in the north. This is also the start of the national epidemic period. The peak was reached in week 9001 (the first week of 1890). In this week there were also 11 cases in the city of Umeå, 100 kilometers north of the railroads at that time. In week 9002 there were 2 cases in Pajala, north of the Arctic Circle near the Finish border. When this northern area peaked in January the influenza was more or less over in the southeastern parts of the country. When our data series ends in week 9009 (last week of February, 1890) the influenza showed high incidence levels only in the northernmost region.

**Figure 5:**
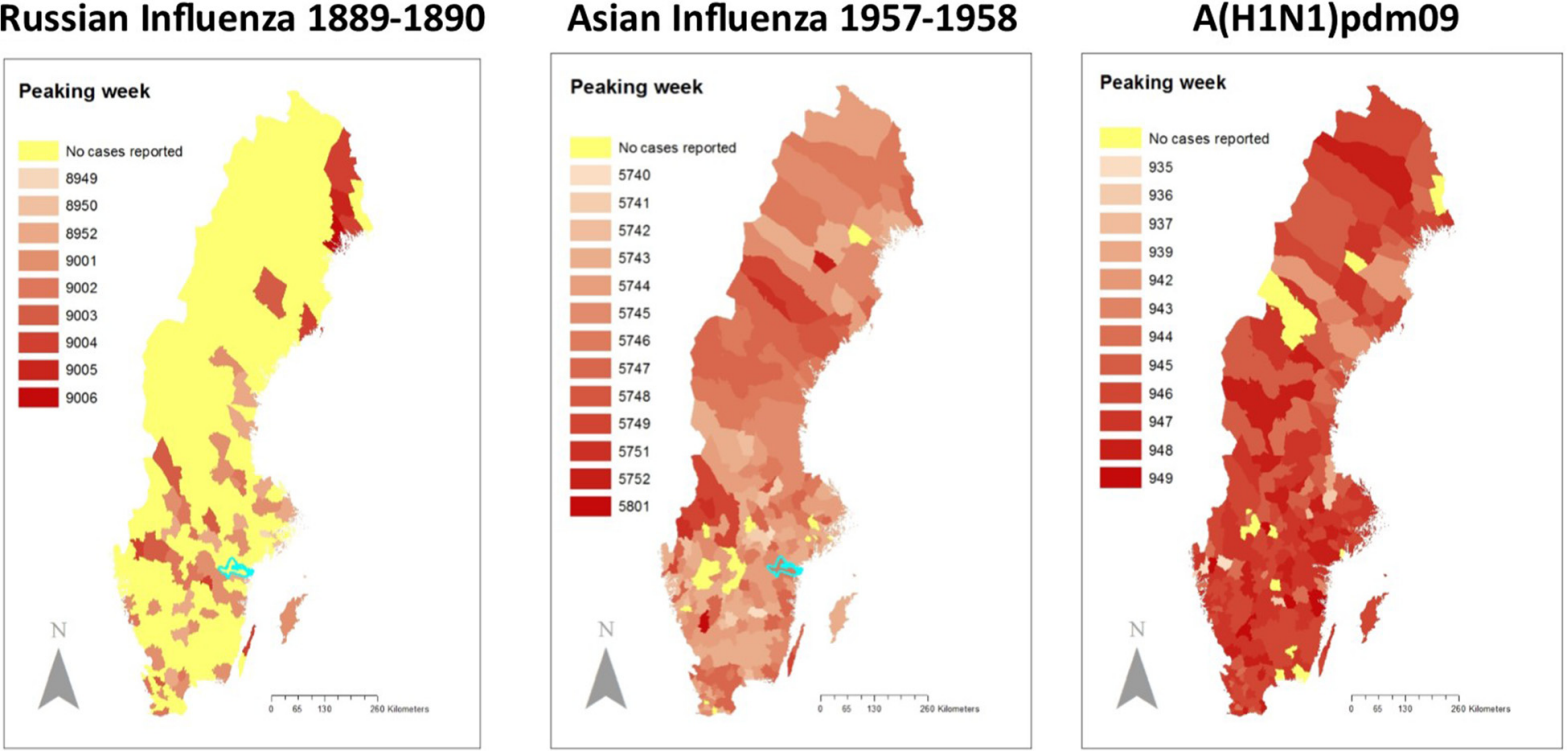
**Peaking week for all municipalities with cases for the three pandemics.** Municipalities represented with darker colours were peaking later than the ones with brighter colours.

For week 9001 (the peak) there was no global autocorrelation when tested with Moran’s I (Moran’s Index: 0. 016466, z-score: −0.041023and p-value: 0.967278). When testing with Ripley’s K-function local clustering could be found with a Kernel distance up to 370 km for week 9001. For weeks outside the epidemic period there was no clustering at all. The number of municipalities with cases is however too small for any meaningful analysis with Moran’s I or Ripley’s K-function.No clustering was found applying Moran’s I on the spatial distribution of peaking weeks, and there was no clear correlation between peaking week and latitude (a map is available in Figure 5).

### Asian influenza

#### Spatiotemporal propagation

In the last week of June 1957 (week 5726) the first 14 cases of the Asian influenza were diagnosed in the county of Gävleborg, 200 km north of Stockholm. In week 5728 the disease was diagnosed in various places from Kristianstad in the South to Sorsele in the North. After 4 weeks (week 5730) the whole country was affected including Gothenburg in the West and Pajala in the North. The number of diagnosed cases was still very low, in total 61 or 0,002% of all diagnosed cases reported.

In week 5740 (the first week of October) there were 1635 (0.6% of total) diagnoses reported. The major epidemic period started week 5739 and the peak of the influenza was reached during the second week of November (week 5743) with 43,290 diagnosed cases (15.7% of the total). At the end of the year (week 5752) the number of diagnosed cases was 4,271 and the last week of January (week 5804) there were 1,705 cases. The reports for the first week of February 1958 (week 5805) contain only 89 diagnosed cases and thereafter the reporting stopped.The spatial distribution of incidence values for municipalities in week 5743 (the peak of the influenza) showed no global autocorrelation when tested with Moran’s I (Moran’s Index: 0.096895, z-score: 1.612274 and p-value: 0.106902). When the spatial distribution was tested with Ripley’s K-function (99 permutations) for the same week local clustering could be found with a Kernel distance up to 350 km. For weeks outside the epidemic period there was no clustering at all. Moran’s I on peaking weeks for all municipalities showed no clustering and there was no clear correlation between peaking week and latitude (a map is available in Figure 5).

#### Climate observations for the Asian influenza

**Figure 6:**
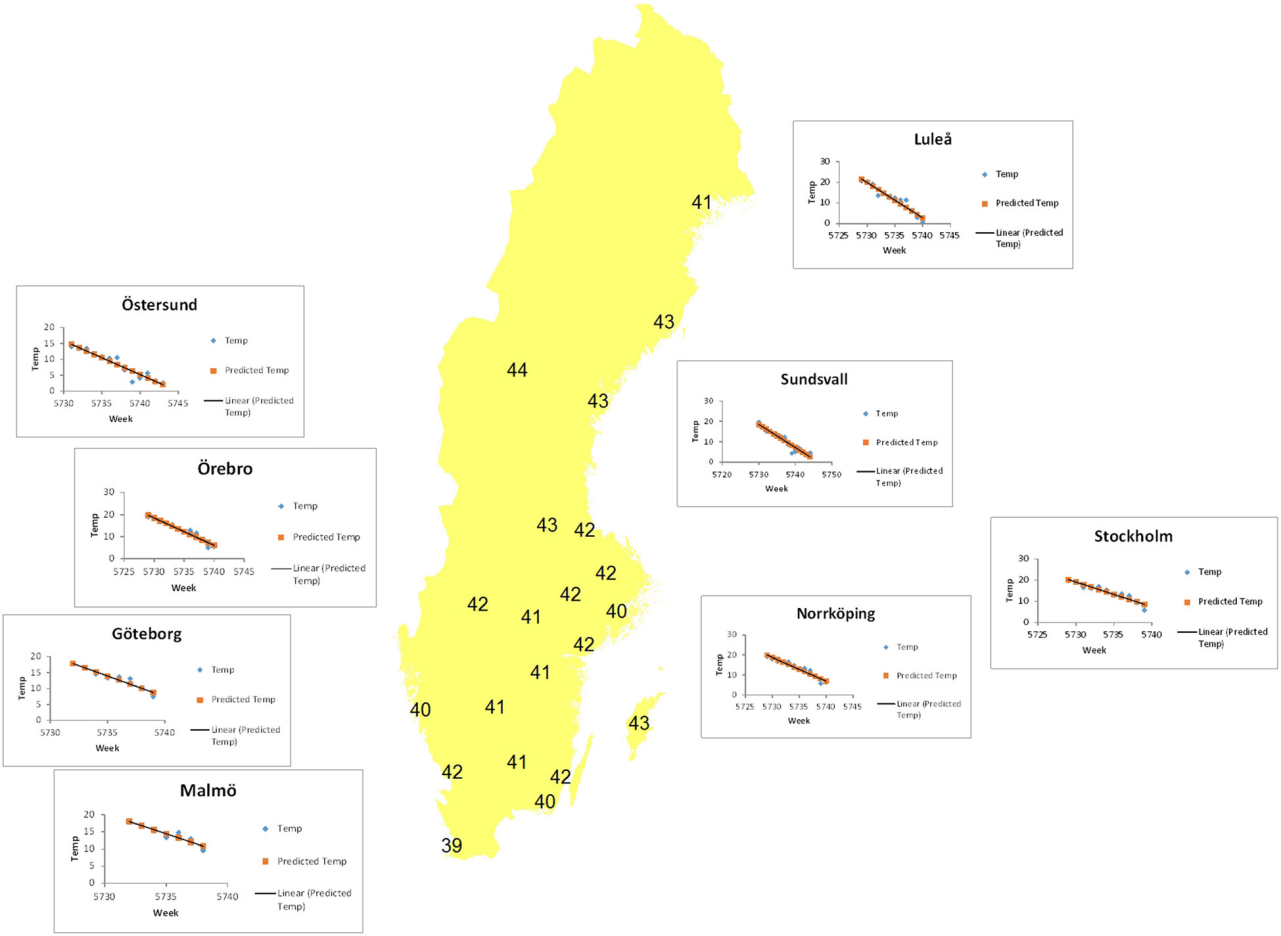
**Onset of pandemic period at county capitals and temperature prior to onset.** The map shows time for onset of pandemic period at all Swedish county capitals. Diagrams depicting temperature fall many weeks prior to the onset are attached for 8 major cities representing 24.4% of the Swedish population in 1957. Weather stations are located at these cities. Norrköping and Sundsvall are not county capitals, but the major cities in their respective counties. Regression statistics are available in Table 2.

**Figure 7:**
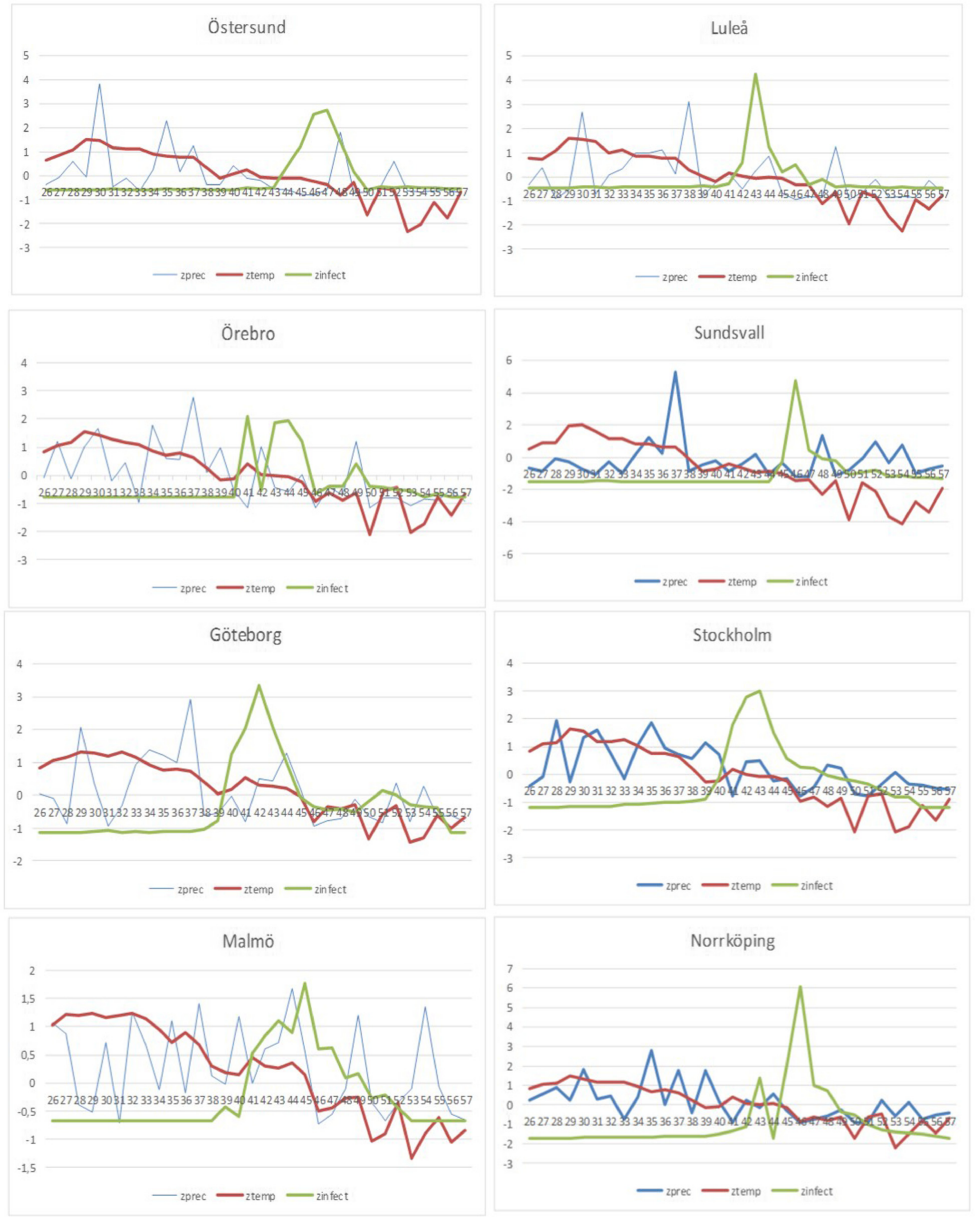
**Temperature, precipitation and number of cases for locations with weather stations.** The diagrams show weekly z-score values ((observation-mean value)/standard deviation) for mean temperature (red graph) and number of Asian Influenza cases (green graph) during 1957–1958. The time series starts with week 5726 (week 1 in the diagrams) and ends with week 5805 (week 31 in the diagrams).

From the least squares linear regression analysis of temperature prior to the onset of the epidemic periods, presented in Figure 6, there is a clear indication that the epidemic period was preceded by falling temperature. Statistics of the regression analysis are available in Table 2. The number of cases in the 8 major cities, where weather stations were located, increased significantly after a long lasting (7 to 15 weeks) local drop of temperature (Figure 7). The temperature at the onset of the epidemic period was between 0.8 and 9.6 °C (colder in the north and warmer in the south) at the locations studied. Precipitation varied irregularly. A correlation between the number of cases and the interpolated weekly data on temperature and precipitation was not found. The maps in Additional files 5 and 6 show the weather situation for week 44 in 1957.

**Table 2:** Least squares linear regression analysis of temperature prior to onset of epidemic periods

	**Malmö**	**Göteborg**	**Norrköping**	**Örebro**	**Stockholm**	**Sundsvall**	**Östersund**	**Luleå**
Multiple R	0,931851	0,955391	0,9616	0,97235	0,94612	0,959574	0,948408	0,961206
R Square	0,868346	0,912772	0,924675	0,945468	0,895144	0,920782	0,899478	0,923917
Adjusted R Square	0,842015	0,898234	0,917142	0,940015	0,883493	0,914688	0,890339	0,916308
Standard Error	1,102432	1,069101	1,278939	1,124103	1,405386	1,547465	1,443481	1,861098
Weeks (n)	7	8	12	12	11	15	13	12
P-value	0,002244	0,000215	6,16E-07	1,21E-07	1,06E-05	1,56E-08	8E-07	6,48E-07
Temp. drop from	17,9	18	19,2	19	19,7	19,7	14	20,8
Temp. drop to	9,6	7,4	6,4	5,6	5,9	4,4	2,6	0,8
Total drop	8,3	10,6	12,8	13,4	13,8	15,3	11,4	20

The table shows statistics from the regression analyses performed on the relation between temperature and time for the weeks prior to epidemic period for 8 major Swedish cities.

Skog et al.

#### Influenza A (H1N1)2009pdm2009-2010

The first laboratory confirmed case of the Influenza A(H1N1)pdm09 was reported close to Gothenburg in the first week of June 2009 (week 0923), followed by 8 cases in Stockholm the week thereafter (Before mandatory reporting including full identity was introduced, two cases were reported from the Stockholm area. The first was hospitalized on April 29, 2009). In week 0925 there was one case in Kristianstad in the south of Sweden. In week 0929 there was also a case in Umeå in the north and in week 0932 (the first week of August) the influenza had reached the city of Boden. The influenza was thereby seeded over the whole country without any major outbreaks. In week 0940 a high number of cases was reported in Skellefteå in the north. The epidemic period started in week 0941. During weeks 0941 and 0942 outbreaks were reported from all over the country. The maximum was reached in week 0946. In week 0950 the epidemic period was over and at the end of 2009 only a few new cases were registered.The statistical evaluation of the spatial distribution of incidence values for municipalities showed patterns similar to those found for the Asian Influenza. For week 0947 there was no global autocorrelation when tested with Moran’s I (Moran’s Index: 0.0492795, z-score: 2.767462 and p-value: 0.005649). When testing with Ripley’s K-function local clustering could be found with a Kernel distance up to 350 km for the weeks 0946 to 0949. For weeks outside the epidemic period there was no clustering at all. When applying Moran’s I on peaking weeks the z-score of 1.66 (p-value: 0.14), indicates a random pattern. As for the other two pandemics, there is no clear correlation between peaking week and latitude (a map is available in Figure 5).

#### GWM for incidence

**Figure 8:**
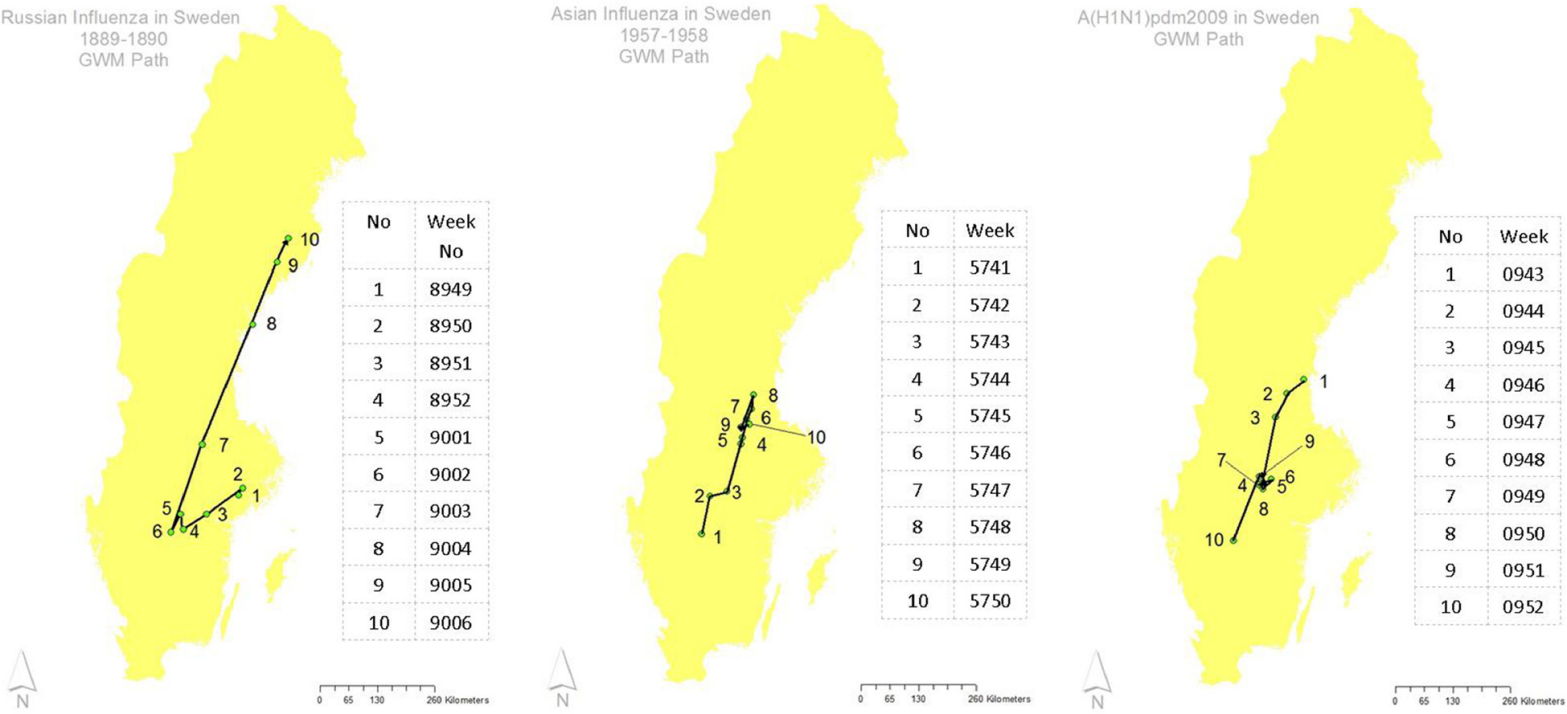
**GWM for incidence.** The three maps depict the path of the GWM (geographic weighted mean) of the incidence per municipality for the 10 weeks epidemic of the Russian Influenza 1889–1890 (left), the Asian Influenza 1957–1958 (middle) and the A(H1N1)pdm2009-2010 (right).

In Figure 8 we have mapped the paths of the GWM for the epidemic periods of the three pandemics. The GWM was found to move along a line from southwest to northeast for the Russian and the Asian influenzas (The GWM for the Russian Influenza moved in the opposite direction in the first weeks of the epidemic period, however with incidence values much lower than those for the rest of the epidemic period). The GWM for the A(H1N1)pdm09 started in the northeast and ended in the southwest. As can be seen in Table 3 the statistical mean direction for the GWM movements of the Asian Influenza and of the Russian Influenza (first three weeks excluded) were both close to northeast. The mean direction for the GWM movements of the A(H1N1)pdm09 was close to southeast.

**Table 3:** Directions of GWM during epidemic period

**Pandemic**	**Compass angle of resulting vector**	**Directional mean (Clockwise from due North)**	**Circular variance**	**Average X (km)**	**Average Y (km)**	**Average length of vector (km)**
Russian	14	75	0.35	581	6751	109
Asian	29	61	0.30	534	6670	50
A(H1N1)pdm09	205	245	0.58	521	6640	59

The table lists the compass angle of the resulting vector from the weekly movements of the GWM for the three pandemics, the mean direction of the individual vectors, their circular variance (Circular variances range from 0 to 1), the coordinates for the mean center and the average length of the weekly movements.

Skog et al.

## Discussion

The pandemics studied in this paper have been discussed in numerous articles on epidemiology, but they have never been analyzed in a common spatiotemporal framework. Using spatial analysis we have been able to map the spread in Sweden of these three influenza pandemics, which have been appearing over a time span of 120 years. During this period infrastructure and social behavior have changed dramatically. The population maps in Additional file 7 show the changes in the distribution of population over these 120 years. Despite those changes we have identified some common patterns of spread, in particular regarding the epidemic period, which lasted approximately 10 weeks for all three pandemics. For obvious reasons influenza reporting has never been and can never be complete, and it never provides more than an estimate of the actual spread.

During the 120 years between the pandemics studied different methods of data collection (case reporting) have been used. We have no means to evaluate the accuracy and completeness of the reporting. However, the high and geographically widespread participation in the post-pandemic study of the Russian Influenza indicates high data completeness. A national system for district physicians for reporting infectious diseases including influenza was established in 1911, and the large number of reporting physicians in 1957 indicates completeness. The reporting by district physicians was abolished in 1989. ILI-reporting, which was used after 1989, has not been functioning well. Instead laboratory verified reports have been evaluated and now they are the leading indicator for influenza surveillance in Sweden [32]. The inclusion of laboratory verified influenza A(H1N1)pdm09 among reportable diseases and a well-established web-based reporting system ensured next to complete reporting from the laboratories. A reference PCR-method was further used for diagnosis and quality control was organized. However, sampling may still have varied. The ratio of positive samples to the number of examined specimen ranged from 0.37 to 0.76 in the 17 of 21 regions that delivered denominators. The highest ratios were reported from the northern and western part of the country. The geographic differences are more likely explained by vaccination and climate than sampling. Sweden offered the pandemic vaccine Pandemrix® to the whole population. The vaccine arrived simultaneously with the onset of the epidemic period, too late to have a profound effect on the spread [14]. The vaccine arrived in weekly batches that were evenly distributed over the country. The pandemic peaked first in the north, and therefore vaccinations may have affected mainly the activity in the southern part of the country. A milder climate in the south may also have diminished the spread. Measures that might have had a general influence on the spread of the pandemics were not recommended in either 1898 or 1957.

The use of incidence is a good measure to describe the spread of a single influenza or a pandemic - thus we believe that the data are rather reliable for the individual pandemics. Incidence per se cannot be used to compare different pandemics with different reporting systems. We believe that the method we developed allows for the comparison of time series of different influenza outbreaks, despite the differences in reporting systems and number of reported cases.

The very high proportion of cases within the epidemic period for the Russian Influenza may partly be explained by lack of data prior to and after the epidemic period. The first known cases were reported in the middle of November, and already in December “it had acquired an epidemic character” [9]. The railroad network played an important role in the dissemination of the Russian Influenza. By 20 December, approximately one month after the first case, the number of places with reported cases was almost twice as high for locations with a railway station as for those without a railway station [10]. The municipalities in the north, not connected by the railroad network, were affected much later than the municipalities in the south and middle of Sweden. Apart from the communications, weather conditions by the end of November were probably very favorable for an immediate spread of the Russian Influenza.

The incidence map animations in general, and the movement of GWM in particular, show that even though the first cases appeared in quite different places for all three pandemics there are similarities. For two of them the GWM moved along a line directed from southwest to northeast. The third moved along a parallel line, but in the opposite direction. A possible explanation for the movements along these lines can be found studying the overall geographic orientation of Sweden and the population maps in Additional file 7. We performed directional distribution (standard deviational ellipse) analyses in ArcGIS to confirm that the major axis of the standard ellipse from Swedish municipalities is rotated 16.5 degrees East (clockwise from noon). A two standard deviations ellipse was used covering approximately 95 percent of the features (municipality centroids) in the cluster. With municipality population for each of the three pandemics added as weight factor the rotation of the major axis increased over the years as can be found in Table 4.

**Table 4:** Directional trends for Swedish municipalities

**Features**	**Short axis (km)**	**Long axis (km)**	**Rotation (degrees clockwise from noon)**
Municipalities (no weight factor)	274,869	894,632	16.5
Municipalities with population 1890 as weight factor	268,830	869,145	14.6
Municipalities with population 1957 as weight factor	281,462	830,222	19.0
Municipalities with population 2009 as weight factor	280,916	769,970	22.4

The table shows the compass angle of the long axis of the standard deviational axis for Swedish municipalities. Population is added as weight factor for the years 1890, 1957 and 2009.

Skog et al.

Charland et al. [33] showed that the timing of the epidemic periods for the annual influenza in cities in the United States was positively correlated to latitude. Chowell et al. [2] made similar observations for the A(H1N1)pdm09 influenza pandemic in Chile, noting that the southern regions experienced earlier pandemic activity than the northern ones. The spread of the Russian Influenza, first appearing in a nation-wide winter climate, was determined by social contact and structure, with the railway being an efficient means of spread. In todays society with almost daily contact facilities covering the whole country, climate may be of greater importance. The similarities in seeding, time of onset and duration of the main peak between the Asian Influenza and the A(H1N1)pdm09 are surprising in view of the rather dramatic changes in contact pattern that have taken place since 1957. However, already in 1889 it was demonstrated that the arrival of one infected person at a new location was sufficient to create a local epidemic [9]. The increased number of contacts today compared to 1957 may therefore be of limited importance for national spread of a pandemic influenza since the virus was already seeded nation-wide before the onset of peaking activity, both in 1957 and in 2009.

The saying that influenza “is spread with the speed of man” seems very true, but the onset of epidemic spread seems to depend on many factors and climate may be one. Atchison et al. [34] found a direct relationship between cold weather and rotavirus transmission in Great Britain and The Netherlands. Shaman et al. [35] calculated vapor pressure (absolute humidity) from relative humidity and temperature data. They showed that the onset of increased wintertime influenza-related mortality in the United States is associated with abnormally low absolute humidity levels during the weeks preceding the onset. When absolute humidity is low, influenza virus survival and transmission is high. Experiments with guinea-pigs have shown that airborne transmission of influenza is facilitated by low temperatures [7]. Chowell et al. [1] showed similar results in studying the propagation of the A(H1N1)pdm09 in New Zealand where the influenza spread in a climate-dependent pattern. Åman [36] reported similar behavior of the Spanish influenza.

Our climate analysis points in the same direction; falling temperature is of importance for the onset of epidemic spread of a seeded influenza. The seeding of the influenza in all parts of Sweden over the summer period, followed by epidemic spread first in October for both the Asian Influenza and the A(H1N1)pdm09 indicates that lowering of temperature is vital for the onset of epidemic spread. The level of precipitation does not seem to have affected the spread of the Asian influenza. Whether the decrease in absolute humidity that accompanies temperature fall is the real reason cannot be verified in this study since information on humidity was not registered for the years 1957–58. However, our results clearly support previous studies that point to climate as an important factor behind influenza spread, and further studies and focus on climate factors are warranted for modeling and planning in the face of a new pandemic.

A major strength of this study is that we have been able to calculate incidence per municipality for our analyses. Census data is not always available and historic population data is generally difficult to obtain in many countries. In Sweden census data and historic population data are easily available and that has been of utmost importance to this work. The main weakness, but also the strength of the study is the data on influenza cases. Access to such detailed data from the same country and for three different pandemics is unique, even though there are limitations in all three data sets. Despite the limitations we believe that the data is sufficiently good to allow for the analysis made. In the end, presenting case data as z-scores of incidence per municipality reduces the shortcomings created by differences in quality and measurement methodology. Our findings shed new light on the spread of pandemic viruses and onset of epidemic periods. These findings may be valuable for future analysis and modeling in the face of a pandemic.

## Conclusions

Looking at the results of the studied pandemics one can conclude that

• Each pandemic had an epidemic period of approximately 10 weeks.

• The weekly geographic weighted mean (GWM) of the incidence of the Russian and the Asian Influenza pandemics is mainly moving along a line from southwest to northeast. The weekly GWM for the A(H1N1)pdm09 influenza pandemic moved in the opposite direction, but along a line parallel to the one above.

• The epidemic period of the Asian Influenza was preceded by falling temperatures.

• The power of spatiotemporal analysis and modeling has been clearly demonstrated and epidemiologists have been given a new tool that can be used to develop new models for preparedness, prediction and analysis.

## Abbreviations

GWM: Geographic weighted mean; A (H1N1) pdm09: Influenza A (H1N1)2009pdm2009-2010; ILI: Influenza-like illness.

## Competing interests

LS is employed by Esri Sverige AB, the Swedish distributor of the GIS software used in the study. LS conducted his PhD studies at the Royal Institute of Technology, Geoinformatics in Stockholm. Except for this, the authors have no competing interests.

## Authors’ contributions

LS designed and carried out the analyses and is the main contributor to the paper. FE is the initiator of the research project. HP was deeply involved in the acquisition of case data from the Asian Influenza. AL contributed significantly to the overall structure of this paper. HH provided advice and guidance during the development of the paper. All authors read and approved the final manuscript.

## Pre-publication history

The pre-publication history for this paper can be accessed here:

http://www.biomedcentral.com/1471-2334/14/378/prepub

## Supplementary Material

Additional file 1 **World wide spread of the Asian Influenza 1957–1958.** An animated map describes the global spread in 1957. Click here for file

Additional file 2 **Spatiotemporal incidence and GWM for the Russian Influenza.** An animated map depicts the propagation in space and time as weekly incidence per municipality along with the geographic weighted mean (GWM) of the incidence numbers. Click here for file

Additional file 3 **Spatiotemporal incidence and GWM for the Asian Influenza.** An animated map depicts the propagation in space and time as weekly incidence per municipality along with the geographic weighted mean (GWM) of the incidence numbers. Click here for file

Additional file 4 **Spatiotemporal incidence and GWM for the A(H1N1)2009pdm2009-2010.** An animated map depicts the propagation in space and time as weekly incidence per municipality along with the geographic weighted mean (GWM) of the incidence numbers. Click here for file

Additional file 5 **Interpolated temperature for week 44 in 1957.** The black dots represent the centroids of all municipalities affected by the Asian Influenza in 1957–1958, whereas the colors represent mean temperature for week 44 in 1957. Click here for file

Additional file 6 **Interpolated precipitation for week 44 in 1957.** The black dots represent the centroids of all municipalities affected by the Asian Influenza in 1957–1958, whereas the colors represent accumulated precipitation (right) for week 44 in 1957. Click here for file

Additional file 7 **Population maps.** The maps depict the distribution of the population at the time of the Russian Influenza 1889–1890 (left), the Asian Influenza 1957–1958 (middle) and the A(H1N1)pdm2009-2010 (right). Each dot represents 1000 people. Click here for file
