# Supplementary material for: Spatiotemporal characteristics of pandemic influenza
Source: BMC Infect Dis. 2014 Jul 9;14:378. doi: 10.1186/1471-2334-14-378 (PMC4226939; doi:10.1186/1471-2334-14-378)
Supplement: Additional file 1 — World wide spread of the Asian Influenza 1957–1958. An animated map describes the global spread in 1957. [file 1471-2334-14-378-S1.zip › Anima11web/ReadMe.html]

xml version="1.0" encoding="utf-8" ?


QuickTime Pro - Exportera för webben - Anima11web


|  |  |
| --- | --- |
|  | Anvisningar för postning av *Anima11web* på din webbplats |

Du har exporterat *Anima11web* med QuickTime Pro. Följande information hjälper dig att posta filmen på din webbplats.

I samma mapp som den här HTML-filen med anvisningar har QuickTime placerat olika versioner av filmen som är optimerade för webbleverans. Det finns även en referensfilm i mappen som väljer bästa version för dina besökares olika enheter och anslutningshastigheter. Genom att lägga in denna referensfilm på HTML-sidan kan du se till att besökare får bästa möjliga videokvalitet på din webbplats.

Här finns en förhandsvisning (kräver Internetanslutning):


Använd följande HTML-kod till att bädda in filmen på webbsidan.

### Viktiga anmärkningar:

- Om dina medier ska kunna visas på Internet måste mediematerialet (filmer, vinjettbild) placeras på en server som alla kan komma åt.
- URL-parametrarna måste ändras om dina mediefiler (filmer, vinjettbilder) ligger på en annan server än din webbsida.
- Filmens URL-parametrar måste alltid peka mot referensfilmen ( Anima11web.mov ) och inte mot källfilmen.
- Referensfilmen måste finnas i samma mapp som källfilmerna.
- Källfilmernas namn får inte ändras, men du kan ändra namn på referensfilmer (kom ihåg att ändra namn i HTML-koden också).
- Om filen ska kunna valideras som XHTML, måste taggen `<style>` och den första `<script>`-taggen finnas i sidans `<head>`-tagg.

Använd den här koden i `<head>` på webbsidan:

<script src="http://www.apple.com/library/quicktime/scripts/ac\_quicktime.js" language="JavaScript" type="text/javascript"></script>
<script src="http://www.apple.com/library/quicktime/scripts/qtp\_library.js" language="JavaScript" type="text/javascript"></script>
<link href="http://www.apple.com/library/quicktime/stylesheets/qtp\_library.css" rel="StyleSheet" type="text/css" />

Använd den här koden i `<body>` på webbsidan:

<script type="text/javascript"><!--
QT\_WritePoster\_XHTML('Klicka här för att spela', 'Anima11web-poster.jpg',
'Anima11web.mov',
'339', '496', '',
'controller', 'true',
'autoplay', 'true',
'bgcolor', 'black',
'scale', 'aspect');
//-->
</script>
<noscript>
<object width="339" height="496" classid="clsid:02BF25D5-8C17-4B23-BC80-D3488ABDDC6B" codebase="http://www.apple.com/qtactivex/qtplugin.cab">
<param name="src" value="Anima11web-poster.jpg" />
<param name="href" value="Anima11web.mov" />
<param name="target" value="myself" />
<param name="controller" value="false" />
<param name="autoplay" value="false" />
<param name="scale" value="aspect" />
<embed width="339" height="496" type="video/quicktime" pluginspage="http://www.apple.com/quicktime/download/"
src="Anima11web-poster.jpg"
href="Anima11web.mov"
target="myself"
controller="false"
autoplay="false"
scale="aspect">
</embed>
</object>
</noscript>

### Exporterat:

- Referensfilm:
  - Anima11web.mov
- Vinjettbild:
  - Anima11web-poster.jpg
- Versioner:
  - Anima11web-desktop.m4v
  - Anima11web-iPhone-cell.3gp
  - Anima11web-iPhone.m4v
