# Supplementary figures and images for: Spatiotemporal characteristics of pandemic influenza
Source: BMC Infect Dis. 2014 Jul 9;14:378. doi: 10.1186/1471-2334-14-378 (PMC4226939; doi:10.1186/1471-2334-14-378)

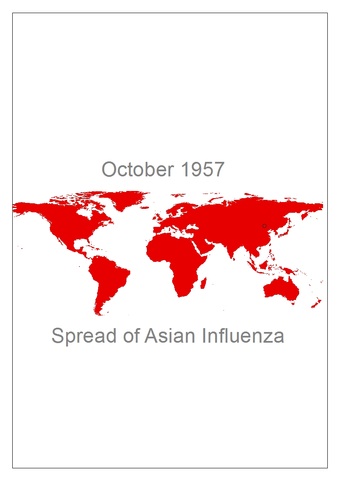

Supplement: Additional file 1 — World wide spread of the Asian Influenza 1957–1958. An animated map describes the global spread in 1957. [file 1471-2334-14-378-S1.zip › Anima11web/Anima11web-poster.jpg]

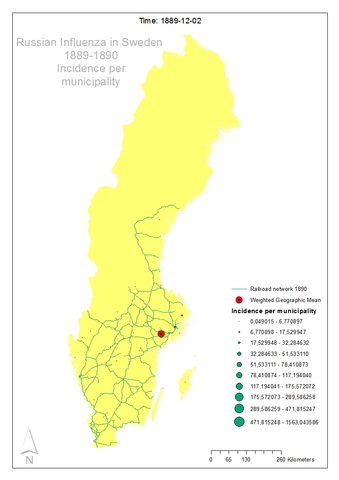

Supplement: Additional file 2 — Spatiotemporal incidence and GWM for the Russian Influenza. An animated map depicts the propagation in space and time as weekly incidence per municipality along with the geographic weighted mean (GWM) of the incidence numbers. [file 1471-2334-14-378-S2.zip › Additional file 2/Additional file 2-poster.jpg]

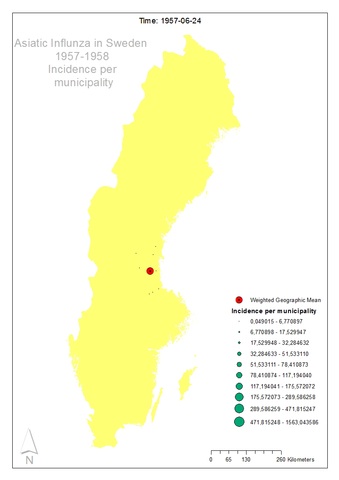

Supplement: Additional file 3 — Spatiotemporal incidence and GWM for the Asian Influenza. An animated map depicts the propagation in space and time as weekly incidence per municipality along with the geographic weighted mean (GWM) of the incidence numbers. [file 1471-2334-14-378-S3.zip › Additional file 3/Additional file 3-poster.jpg]

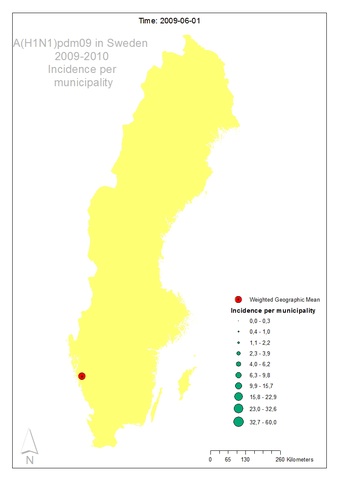

Supplement: Additional file 4 — Spatiotemporal incidence and GWM for the A(H1N1)2009pdm2009-2010. An animated map depicts the propagation in space and time as weekly incidence per municipality along with the geographic weighted mean (GWM) of the incidence numbers. [file 1471-2334-14-378-S4.zip › Anima21web/Anim21web-poster.jpg]

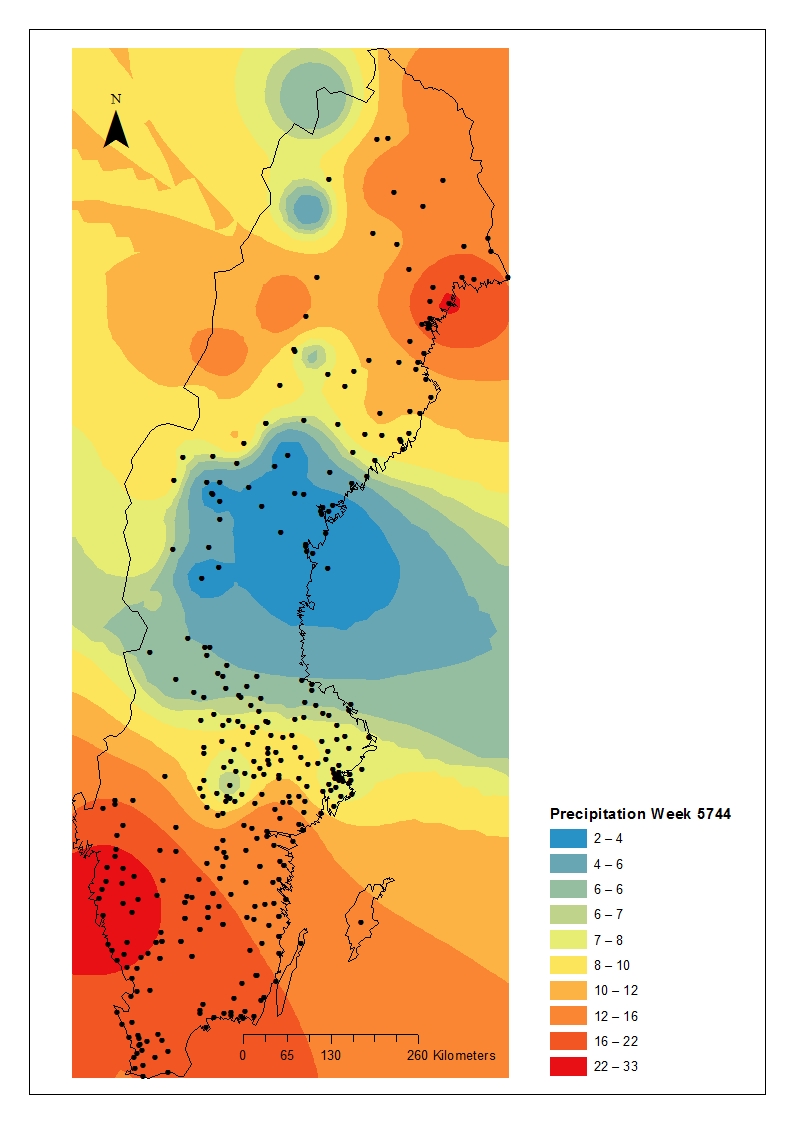

Supplement: Additional file 5 — Interpolated temperature for week 44 in 1957. The black dots represent the centroids of all municipalities affected by the Asian Influenza in 1957–1958, whereas the colors represent mean temperature for week 44 in 1957. [file 1471-2334-14-378-S5.jpeg]

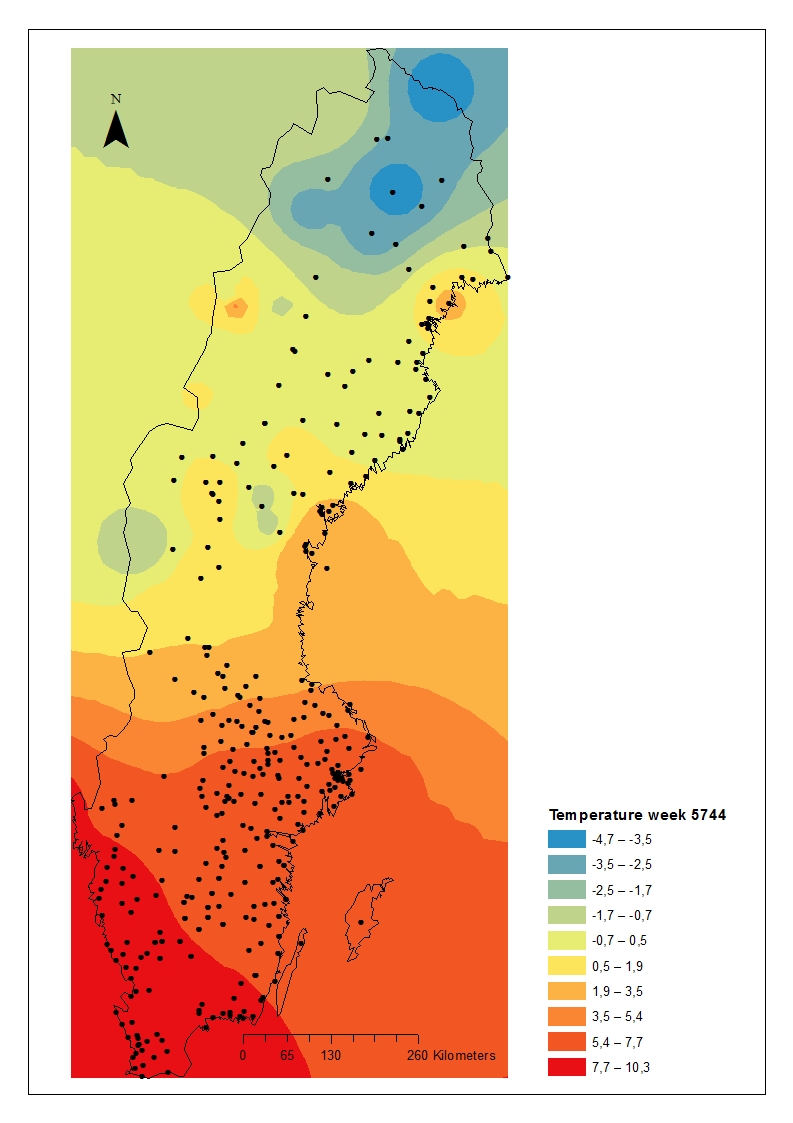

Supplement: Additional file 6 — Interpolated precipitation for week 44 in 1957. The black dots represent the centroids of all municipalities affected by the Asian Influenza in 1957–1958, whereas the colors represent accumulated precipitation (right) for week 44 in 1957. [file 1471-2334-14-378-S6.jpeg]

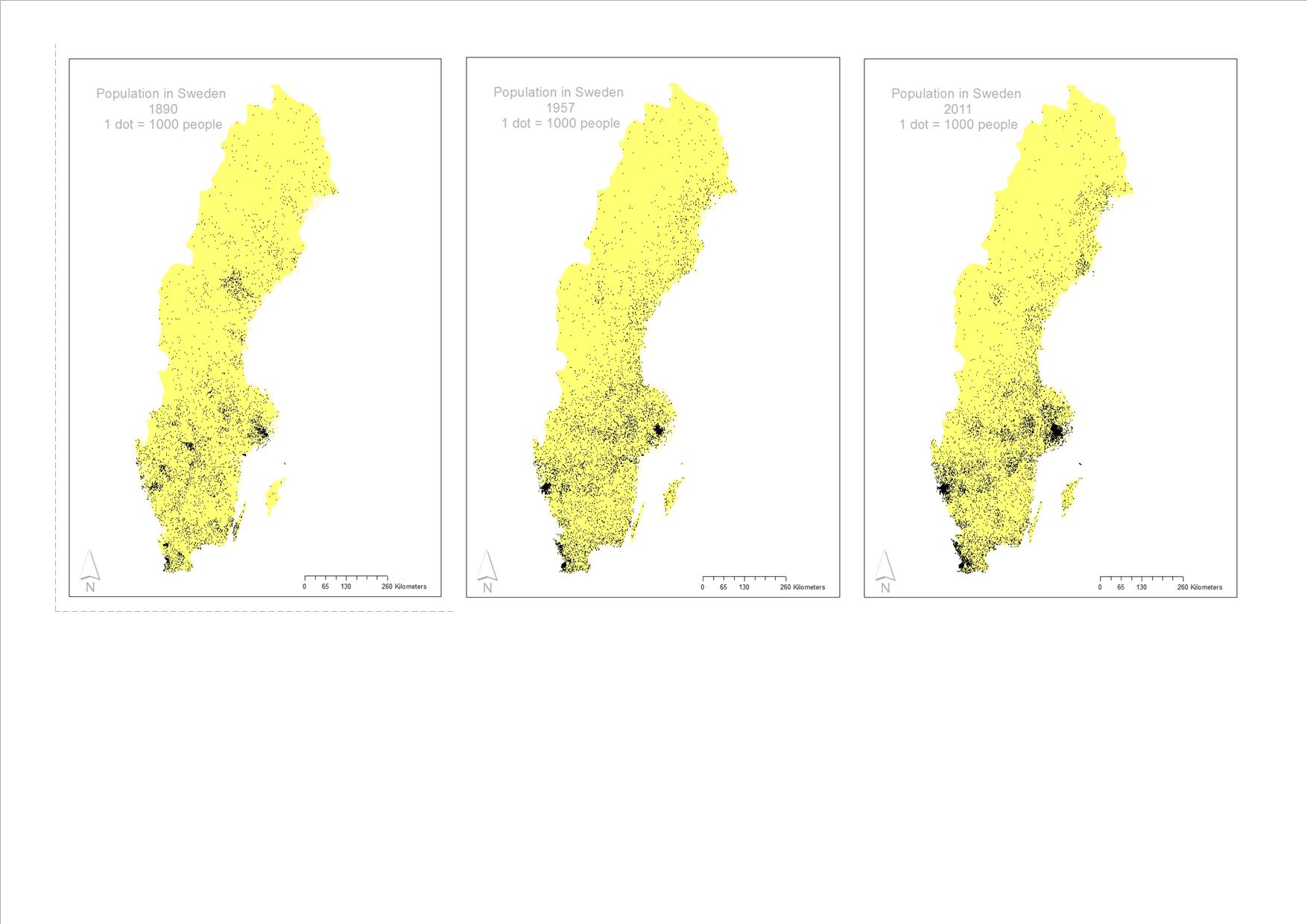

Supplement: Additional file 7 — Population maps. The maps depict the distribution of the population at the time of the Russian Influenza 1889–1890 (left), the Asian Influenza 1957–1958 (middle) and the A(H1N1)pdm2009-2010 (right). Each dot represents 1000 people. [file 1471-2334-14-378-S7.jpg]
