# Supplementary material for: Spatiotemporal characteristics of pandemic influenza
Source: BMC Infect Dis. 2014 Jul 9;14:378. doi: 10.1186/1471-2334-14-378 (PMC4226939; doi:10.1186/1471-2334-14-378)
Supplement: Additional file 2 — Spatiotemporal incidence and GWM for the Russian Influenza. An animated map depicts the propagation in space and time as weekly incidence per municipality along with the geographic weighted mean (GWM) of the incidence numbers. [file 1471-2334-14-378-S2.zip › Additional file 2/ReadMe.html]

xml version="1.0" encoding="utf-8" ?


QuickTime Pro - Exportera för webben - Additional file 2


|  |  |
| --- | --- |
|  | Anvisningar för postning av *Additional file 2* på din webbplats |

Du har exporterat *Additional file 2* med QuickTime Pro. Följande information hjälper dig att posta filmen på din webbplats.

I samma mapp som den här HTML-filen med anvisningar har QuickTime placerat olika versioner av filmen som är optimerade för webbleverans. Det finns även en referensfilm i mappen som väljer bästa version för dina besökares olika enheter och anslutningshastigheter. Genom att lägga in denna referensfilm på HTML-sidan kan du se till att besökare får bästa möjliga videokvalitet på din webbplats.

Här finns en förhandsvisning (kräver Internetanslutning):


Använd följande HTML-kod till att bädda in filmen på webbsidan.

### Viktiga anmärkningar:

- Om dina medier ska kunna visas på Internet måste mediematerialet (filmer, vinjettbild) placeras på en server som alla kan komma åt.
- URL-parametrarna måste ändras om dina mediefiler (filmer, vinjettbilder) ligger på en annan server än din webbsida.
- Filmens URL-parametrar måste alltid peka mot referensfilmen ( Additional file 2.mov ) och inte mot källfilmen.
- Referensfilmen måste finnas i samma mapp som källfilmerna.
- Källfilmernas namn får inte ändras, men du kan ändra namn på referensfilmer (kom ihåg att ändra namn i HTML-koden också).
- Om filen ska kunna valideras som XHTML, måste taggen `<style>` och den första `<script>`-taggen finnas i sidans `<head>`-tagg.

Använd den här koden i `<head>` på webbsidan:

<script src="http://www.apple.com/library/quicktime/scripts/ac\_quicktime.js" language="JavaScript" type="text/javascript"></script>
<script src="http://www.apple.com/library/quicktime/scripts/qtp\_library.js" language="JavaScript" type="text/javascript"></script>
<link href="http://www.apple.com/library/quicktime/stylesheets/qtp\_library.css" rel="StyleSheet" type="text/css" />

Använd den här koden i `<body>` på webbsidan:

<script type="text/javascript"><!--
QT\_WritePoster\_XHTML('Klicka här för att spela', 'Additional%20file%202-poster.jpg',
'Additional%20file%202.mov',
'339', '496', '',
'controller', 'true',
'autoplay', 'true',
'bgcolor', 'black',
'scale', 'aspect');
//-->
</script>
<noscript>
<object width="339" height="496" classid="clsid:02BF25D5-8C17-4B23-BC80-D3488ABDDC6B" codebase="http://www.apple.com/qtactivex/qtplugin.cab">
<param name="src" value="Additional%20file%202-poster.jpg" />
<param name="href" value="Additional%20file%202.mov" />
<param name="target" value="myself" />
<param name="controller" value="false" />
<param name="autoplay" value="false" />
<param name="scale" value="aspect" />
<embed width="339" height="496" type="video/quicktime" pluginspage="http://www.apple.com/quicktime/download/"
src="Additional%20file%202-poster.jpg"
href="Additional%20file%202.mov"
target="myself"
controller="false"
autoplay="false"
scale="aspect">
</embed>
</object>
</noscript>

### Exporterat:

- Referensfilm:
  - Additional file 2.mov
- Vinjettbild:
  - Additional file 2-poster.jpg
- Versioner:
  - Additional file 2-desktop.m4v
  - Additional file 2-iPhone-cell.3gp
  - Additional file 2-iPhone.m4v
